# Inflammation Following Traumatic Brain Injury in Humans: Insights from Data-Driven and Mechanistic Models into Survival and Death

**DOI:** 10.3389/fphar.2016.00342

**Published:** 2016-09-27

**Authors:** Andrew Abboud, Qi Mi, Ava Puccio, David Okonkwo, Marius Buliga, Gregory Constantine, Yoram Vodovotz

**Affiliations:** ^1^Department of Surgery, University of PittsburghPittsburgh, PA, USA; ^2^Department of Sports Medicine and Nutrition, University of PittsburghPittsburgh, PA, USA; ^3^Center for Inflammation and Regenerative Modeling, McGowan Institute for Regenerative Medicine, University of PittsburghPittsburgh, PA, USA; ^4^Department of Neurological Surgery, University of PittsburghPittsburgh, PA, USA; ^5^Department of Mathematics, University of PittsburghBradford, PA, USA; ^6^Department of Mathematics and Department of Statistics, University of PittsburghPittsburgh, PA, USA

**Keywords:** inflammation, TBI outcome, mathematical modeling, data-driven modeling, mortality, inflammatory mediators in CNS

## Abstract

Inflammation induced by traumatic brain injury (TBI) is a complex mediator of morbidity and mortality. We have previously demonstrated the utility of both data-driven and mechanistic models in settings of traumatic injury. We hypothesized that differential dynamic inflammation programs characterize TBI survivors vs. non-survivors, and sought to leverage computational modeling to derive novel insights into this life/death bifurcation. Thirteen inflammatory cytokines and chemokines were determined using Luminex™ in serial cerebrospinal fluid (CSF) samples from 31 TBI patients over 5 days. In this cohort, 5 were non-survivors (Glasgow Outcome Scale [GOS] score = 1) and 26 were survivors (GOS > 1). A Pearson correlation analysis of initial injury (Glasgow Coma Scale [GCS]) vs. GOS suggested that survivors and non-survivors had distinct clinical response trajectories to injury. Statistically significant differences in interleukin (IL)-4, IL-5, IL-6, IL-8, IL-13, and tumor necrosis factor-α (TNF-α) were observed between TBI survivors vs. non-survivors over 5 days. Principal Component Analysis and Dynamic Bayesian Network inference suggested differential roles of chemokines, TNF-α, IL-6, and IL-10, based upon which an ordinary differential equation model of TBI was generated. This model was calibrated separately to the time course data of TBI survivors vs. non-survivors as a function of initial GCS. Analysis of parameter values in ensembles of simulations from these models suggested differences in microglial and damage responses in TBI survivors vs. non-survivors. These studies suggest the utility of combined data-driven and mechanistic models in the context of human TBI.

## Introduction

Traumatic brain injury (TBI) remains a leading medical problem in the United States, causing severe long-term morbidity and, in some cases, mortality. Between 1.6 and 3.8 million sports-related concussions alone are reported each year, while an estimated 5.3 million people are living with long-term cognitive and psychological impairments annually (Selassie et al., 2008). These traumatic injuries are caused by direct and indirect biomechanical forces to the cranium, and result in a neurometabolic energy crisis in the brain (Giza and Hovda, 2014). These changes have immediate pathogenic effects on ion homeostasis, regional cerebral blood flow (CBF), and cerebral metabolism, while also carrying downstream “immunoexcitotoxicity” effects by activating immune receptors on microglia (brain macrophage-like cells) and astrocytes in response to cellular injury and oxidative stress (Blaylock and Maroon, 2011; Woodcock and Morganti-Kossmann, 2013). These cellular and molecular cascades can be both neuroprotective and neurotoxic, and ultimately impact patients' prognosis and recovery (Selassie et al., 2008).

While the clinical progression and biological underpinnings of TBI have been studied extensively, accurately predicting a patient's prognosis following a TBI remains a difficult challenge for clinicians. Traditionally, the diagnosis of concussions and TBI have fallen into a “one size fits all” approach. Recently, however, there has been impetus toward a multifaceted and targeted approach that matches a TBI treatment plan with a number of variable clinical trajectories (Collins et al., 2014). Even so, current predictors of outcome after severe TBI are neither sufficiently sensitive nor specific to be used for clinical decision making in the acute recovery period (Gao and Zheng, 2015).

Thus, although the clinical segregation of TBI patients is improving, there still remains a need for clear biomarkers and/or diagnostic modalities to predict individual patient's likelihood of recovery, disability, and mortality. This challenge is not unique to TBI, but has proven to be an obdurate obstacle for health care providers in the management of many disease states including cancer, sepsis, and transplantation.

One central process that affects outcomes following TBI is that of acute inflammation, involving mediators such as TNF-α, IL-6, IL-8, and IL-10 (Woodcock and Morganti-Kossmann, 2013). However, the inflammatory response to injury and infection is complex, dynamic, and highly dependent on patient-specific conditions. We and others have shown the efficacy of data-driven computational models in segregating patients using circulating inflammatory cytokines, chemokines, and damage-associated molecular pattern (DAMP) molecules (Zaaqoq et al., 2014; Namas et al., 2015; Almahmoud et al., 2015a,b; Abboud et al., 2016; Namas et al., 2016b). We have recently reported on the utility of multiplexed analysis of circulating inflammatory mediators combined with data-driven modeling to define potentially novel inflammatory mechanisms that segregate survivors vs. non-survivors of blunt trauma (Abboud et al., 2016). Separately, mechanistic mathematical models have served as the basis for patient-specific predictions and virtual clinical trials in the setting of human blunt trauma, including an exploration of the inflammatory characteristics of survivors and non-survivors (Brown et al., 2015).

Herein, we hypothesized that early TBI-induced inflammation can foreordain similar patients for survival vs. mortality, and carried out a combined clinical and *in silico* study to gain insights into this process. In the present study, we sought to gain insights from data-driven modeling to develop mechanistic models of TBI, in order to better understand the inflammatory characteristics of TBI survivors and non-survivors.

## Materials and methods

### Traumatic brain injury patients

Severe TBI patients were enrolled prospectively as approved by the University of Pittsburgh Institutional Review Board upon meeting inclusion criteria judged by the on-call neurosurgeon. Informed consent was obtained by the legal authorized representative prior to study procedures. CSF and blood samples were obtained by trained study personnel for the initial enrollment and through 5 days of ICU admission. A trained neuropsychological technician obtained the 6- and 12-month Glasgow Outcome Scale (GOS) scores. The patient cohort consisted of 34 TBI patients [29 survivors (27 males/2 females) and 5 non-survivors (4 males/1 female)]. Non-survivors were determined by having a Glasgow Outcome Scale (GOS) score of 1 by 12 month follow up, and had a Glasgow Coma Scale (GCS) score (an estimate of TBI injury severity) of 5.6 ± 0.58 on hospital arrival. Survivors had a similar admission GCS of 6.2 ± 0.26 (Table 1), with a GOS score of 2–5.

**Table 1:** **General demographics and injury characteristics of TBI patient cohort**.

	**Survivors (*n* = 29)**	**Non-survivors (*n* = 5)**
Age	31.6 ± 5.6	41.0 ± 7.4
Sex ratio (M:F)	27:2	4:1
GCS	6.0 ± 0.24	5.6 ± 0.57

### Clinical data

The data on each subject consisted of two distinct components, namely clinical/demographic data and CSF inflammatory mediator data. Clinical/demographic (one-dimensional) variables included: age, gender, presence of infection, bleeding, surgical decompression, presence of subarachnoid hemorrhage, and initial GCS score. The GCS score quantified the initial brain injury severity on a numerical scale from 3 to 15. The GOS score was utilized as the outcome variable and was viewed as the response variable to study and predict neurological outcome, as a function of the other input variables. The GOS score quantifies the neurological outcome at 6 and 12 months post-TBI. GOS scores ranged from 1 to 5, with 1 indicating death and higher values indicating a progressively better neurological state of health.

### Inflammatory mediator data

In addition to the clinical and demographic data, inflammatory mediator data were collected from all 34 patients. The data were plotted as time series for the following 13 cytokines/chemokines (assayed using Luminex™ multiplexing technology): IL-1α, IL-1β, IL-2, IL-4, IL-5, IL-6, IL-8, IL-10, IL-13, macrophage inflammatory protein (MIP)-1α, MIP-1β, tumor necrosis factor (TNF)-α, and vascular endothelial growth factor (VEGF). The inflammatory mediator time series variables varied in both in length and in the time sequence at which they were collected.

### Statistical analysis

All data were analyzed using SigmaPlot™ 11 software (Systat Software, Inc., San Jose, CA). Statistical difference between survivors and non-survivors was determined by Student's *t*-test. Group-time interaction of plasma inflammatory mediators' levels between survivors and non-survivors was determined by Two-Way Analysis of Variance (ANOVA) and quantified by area under the curve (AUC) using the mean values for each time point, then calculating non-survivors/survivors AUC fold change. Statistical significance for this study was set at a *p* < 0.05.

### Computational methods: PCA

Normalized inflammatory mediator data (each measurement taken to be a single point in 13-dimensional inflammatory mediator space) were transformed into principal component space using the MatLab® function, princomp. We then examined projections of inflammatory mediators into principal component space by using the score coefficients for the first 2 or 3 principal components.

### Computational methods: DyBN

We carried out Dynamic Bayesian Network (DyBN) inference to model the evolution of probabilistic dependencies within a system over time, and to suggest possible feedback interactions among inflammatory mediators. This analysis was carried out using MATLAB™ (The Math Works, Inc., Natick, Ma) as previously described by our group (Azhar et al., 2013; Almahmoud et al., 2015c; Abboud et al., 2016). Inflammatory mediators were represented at multiple time points within the same network structure. The data were separated in adjacent 24 h time periods up to 5 days (0–24 h, 24–48 h…96–120 h). In this approach, time was modeled discretely as in a discrete Markov chain. Each mediator was given a time index subscript indicating the time slice to which it belonged. Additional temporal dependencies were represented in a DyBN by edges between time slices. Each node in the network was associated with a conditional probability distribution of a variable that is conditioned upon its parents (upstream nodes).

### Computational methods: ordinary differential equation model

We constructed a mechanistic model using ordinary differential equations (ODE), based on core interactions inferred from PCA and DyBN. Before constructing this model, we sought to gain further insights into potential relationships between time and peaks in the CSF levels of these cytokines. Accordingly, the distributions of Peak time of TNF-α, IL-6, and IL-10) were plotted. This analysis suggested that most patient's TNF-α and IL-10 peak at the early time point (*t* < 20 h post-injury) and IL-6 peaks slightly later (at ~30 h post-injury).

The model equations are depicted below:

$$\begin{array}{l} \;\frac{d\text{D}}{d\text{t}}={\text{d}}_{0}\text{M}-{\text{d}}_{1}\text{D}\\ \;\frac{d\text{M}}{d\text{t}}=\left(\text{​}\frac{{\text{m}}_{0}\text{D}}{1+{\text{m}}_{1}\text{D}}+\frac{{\text{m}}_{2}\text{C}}{1+{\text{m}}_{3}\text{C}}+\frac{{\text{m}}_{4}\text{TNF}}{1+{\text{m}}_{5}\text{TNF}}+\frac{{\text{m}}_{6}{\text{IL}}_{6}}{1+{\text{m}}_{7}{\text{IL}}_{6}}\text{​}\right)\\ \;\frac{1}{1+{\text{m}}_{8}{\text{IL}}_{10}}-{\text{m}}_{9}\text{M}\\ \;\frac{d\text{C}}{d\text{t}}=\frac{{\text{c}}_{0}\text{D}}{1+{\text{c}}_{1}\text{D}}-{\text{c}}_{2}\text{C}\\ \frac{d{\text{IL}}_{10}}{d\text{t}}=i_{0}\text{M}-i_{1}{\text{IL}}_{10}\\ \frac{d\text{TNF}}{d\text{t}}={\frac{{\text{t}}_{0}\text{M}}{1+{\text{t}}_{1}\text{IL}}}_{10}-{\text{t}}_{2}\text{TNF}\\ \frac{d{\text{IL}}_{6}}{d\text{t}}={\frac{{\text{b}}_{0}{\text{M}}^{6}}{1+{\text{b}}_{1}\text{IL}}}_{10}-{\text{b}}_{2}{\text{IL}}_{6} \end{array}$$

Terms in the model: Damage/dysfunction/DAMPs (D), Activated inflammatory cells/microglia (M), Chemokine (C), IL-10 (IL_10_), TNF-α (TNF), IL-6 (IL_6_).

This model encompasses the following biology. Cytokines and chemokines are produced by inflammatory cells, which in turn are activated by damage (not directly) and further production of cytokines and chemokines. Model variables and equations are the following: damage (D), inflammatory cells (M), chemokine (C), TNF-α, IL-6, and IL-10. This model incorporates positive feedback from the pro-inflammatory cytokines TNF-α and IL-6, and negative feedback from the anti-inflammatory cytokine IL-10.

The damage variable D is a lumped variable that stands both for overall tissue damage/dysfunction (e.g., changes in CBF, elevated intracranial pressure, and associated neurometabolic changes (Blaylock and Maroon, 2011; Woodcock and Morganti-Kossmann, 2013; Giza and Hovda, 2014), and, at a molecular level, the DAMPs released from damaged or dysfunctional brain tissue (e.g., S100B; Woodcock and Morganti-Kossmann, 2013). This variable is assumed to be driven only by activated inflammatory cells/microglia (M), and regulated negatively by both anti-inflammatory influences as well as self-decay. The most important equation in the model is the one for inflammatory cells (M), because the level of these cells regulates the level of all cytokines in this model.

Model parameters and their biological correlates are depicted in Table 2. Before fitting our models to CSF data from TBI patients, the actual data including cytokines and GCS (which represents the initial damage value was normalized to a scale from 0 to 10. In the case of damage, an initial damage value of zero is equated with a GCS score of 15, and an initial damage value of 10 represents a GCS score of 3. For cytokines, the mean cytokine value in survivors and non-survivors were calculated at time points: 0, 17, 35, 60, 82, and 104 h. In addition to the model parameters shown in the equations (see above), two additional parameters (the initial values of inflammatory cells and chemokine) had to be estimated.

**Table 2:** **Parameters of ordinary differential equation model of TBI**.

**Parameter name**	**Biological function of parameter**
d_0_	Rate of damage/DAMP production by activated inflammatory cells/microglia
d_1_	Decay rate of damage/DAMPs
m_0_	Rate of inflammatory cell activation in response to damage/DAMPs
m_1_	Rate of inflammatory cell inhibition in response to damage/DAMPs
m_2_	Rate of inflammatory cell activation in response to chemokines
m_3_	Rate of inflammatory cell inhibition in response to chemokines
m_4_	Rate of inflammatory cell production in response to TNF-α
m_5_	Rate of inflammatory cell Inhibition in response TNF-α
m_6_	Rate of inflammatory cell activation in response to IL-6
m_7_	Rate of inflammatory cell inhibition in response to IL-6
m_8_	Degree to which IL-10 inhibits the activation of inflammatory cells
m_9_	Death rate of Inflammatory cells
c_0_	Rate of chemokine production induced by damage/DAMPs
c_1_	Degree to which damage/DAMPs inhibit chemokine release
c_2_	Decay rate of chemokines
i_0_	Rate of IL-10 production by activated inflammatory cells
i_1_	Decay rate of IL-10
t_0_	Rate of TNF-α production by inflammatory cells
t_1_	Degree to which IL-10 inhibits TNFα
t_2_	Decay rate of TNF-α
b_0_	Rate of IL-6 production by inflammatory cells
b_1_	Degree to which IL-10 inhibits IL-6
b_2_	Decay rate of IL-6
init_C	Initial value for chemokines
Init_M	Initial value for inflammatory cells

Our fitting procedure was as follows. We choose the initial guess for parameters (randomly or based on previous parameter estimations) and applied the Nelder-Mead simplex method for parameter optimization. The error function in our case is just the sum of errors between computed values of cytokines from the model equations and the values from original mean cytokine values (we simply computed the Euclidean distance between two data vectors). All analysis was performed using Matlab®.

## Results

### TBI survivors and non-survivors are not be segregated by glasgow coma scale score, suggesting divergent responses to injury

**Figure 1:**
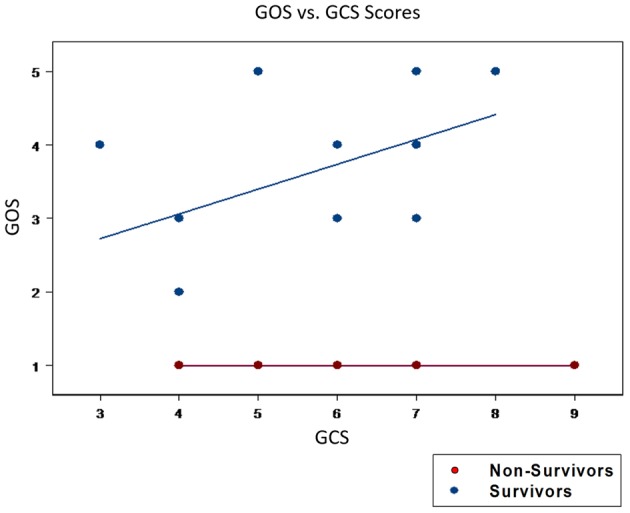
**Initial GCS scores could not segregate patients for mortality vs. survival**. An initial GCS score was calculated following the traumatic insult. The GOS score was utilized as the outcome variable and was viewed as the response variable. The GOS score quantifies the neurological outcome at 6 and 12 months post-TBI. GOS scores ranged from 1 to 5, with 1 indicating death and higher values indicating a progressively better neurological state of health. There were no statistical differences in the mean GCS between survivors (6.0 ± 0.24) and non-survivors (5.6 ± 0.57, *p* = 0.313). Furthermore, a plot of GCS vs. GOS was unable to cluster those who went onto survive (GOS > 1) from those who died (GOS = 1). These findings highlight the inutility of GCS alone in predicting patient outcomes.

The overall enrollment resulted in a final cohort of 29 survivors (mean age: 31.6 ± 5.6) with a GCS of 6.0 ± 0.24. Five non-survivors (mean age: 41.0 ± 7.4, *p* = 0.172) with a GCS of 5.6 ± 0.57, *p* = 0.313 (Table 1). Thus, there were no significant differences between groups in age or GCS. A comparison between groups of initial GCS with a 6 month GOS per patient reveals no apparent correlation between the severity of the initial head trauma with 6 month survival (GOS > 1) and mortality (GOS = 1; Figure 1). These results suggest that patient-specific factors in response to TBI, rather than magnitude of injury alone, drive differential outcomes following injury.

We therefore hypothesized that TBI-induced cerebral inflammation drives, or is associated with, the divergent outcomes of survival and death following TBI. To test this hypothesis, we analyzed the dynamics of multiple inflammatory mediators in the CSF, using both standard statistical analyses and computational modeling.

### Time courses of CSF inflammatory mediators segregate survivors from non-survivors

**Figure 2:**
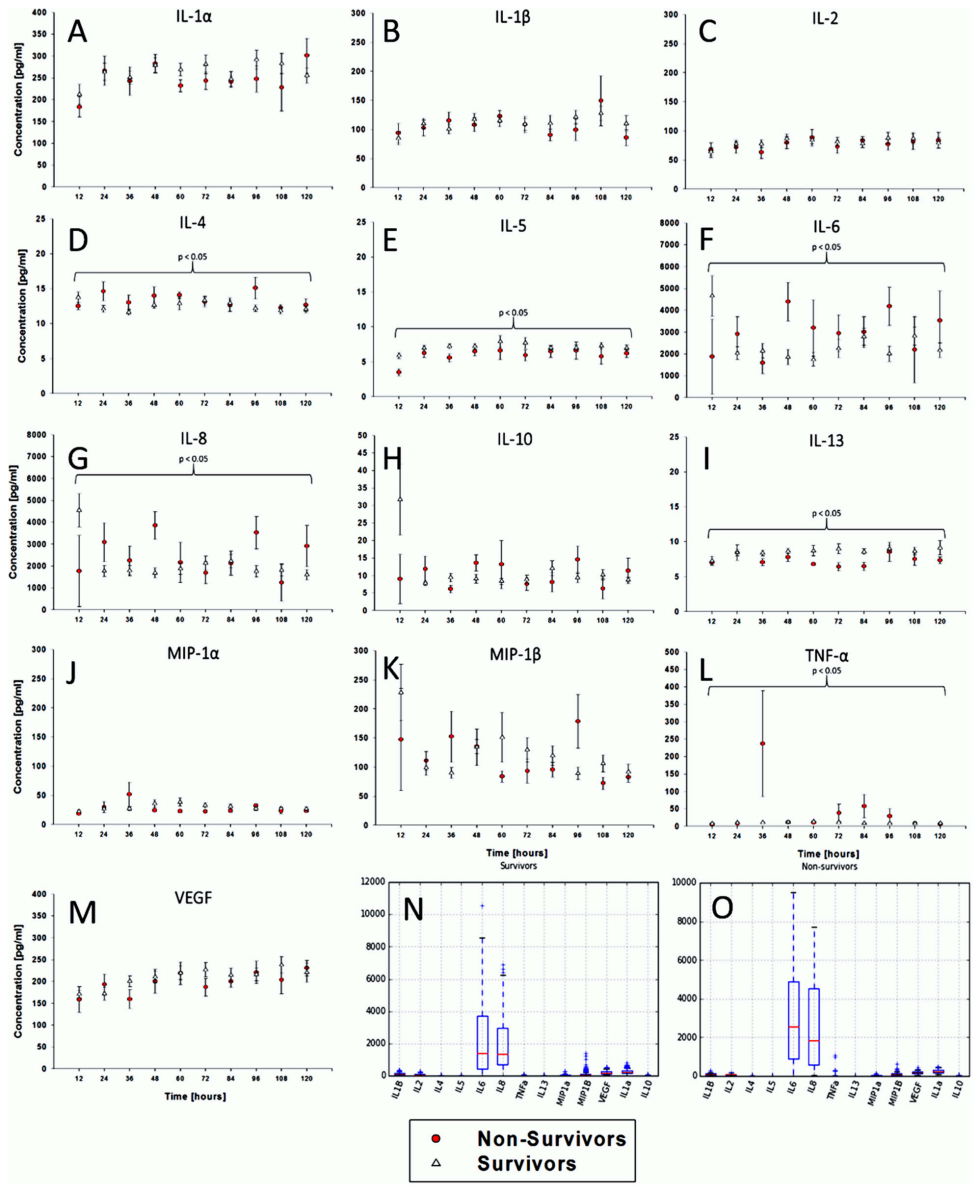
**Time courses of CSF inflammatory mediators segregate survivors from non-survivors**. In addition to the clinical and demographic data, inflammatory mediator data were collected from all 34 patients. The data were plotted as time series for the following 13 cytokines/chemokines. The inflammatory mediator time series variables varied in both in length and in the time sequence at which they were collected. **(A–M)** Time course analyses for each of 13 inflammatory mediators revealed differences in 6 mediators over 5 days by Two-Way ANOVA, IL-4, 5, 6, 8, 13, and TNF-α. The mediator levels were highly variable, oscillating frequently and at relatively low levels. **(N,O)** Notably, levels of IL-6 and IL-8 were the only mediators with mean values elevated above 1000 pg/ml.

We first hypothesized that dynamics of CSF inflammatory mediators, as a surrogate for brain inflammation following TBI, would differ between survivors and non-survivors. In support of this hypothesis, time course analyses for each of 13 inflammatory mediators revealed differences in 6 mediators over 5 days by Two-Way ANOVA, IL-4, 5, 6, 8, 13, and TNF-α (Figures 2A–M). Notably, levels of IL-6 and IL-8 were the only mediators with mean values elevated above 1000 pg/ml (Figures 2N,O).

### Principal component analysis suggests conserved general wiring of inflammation in TBI survivors and non-survivors

**Figure 3:**
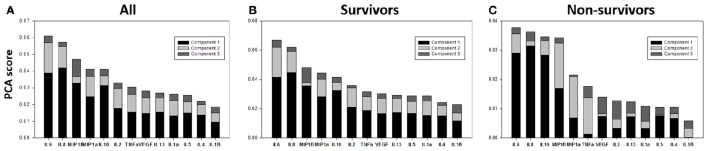
**Survivors and non-survivors share a similar core inflammatory wiring. (A)** PCA analysis was carried out on survivor and non-survivor data combined to assess the underlying inflammatory process. This analysis identified a linear combination of TNF-α, MIP-1α, MIP-1β, IL-8, IL-10, and IL-6 as part of the first principal component. **(B,C)** We next carried out this analysis separately on survivors and non-survivors, with the null hypothesis being that there would not be any significant differences between these groups based on PCA if the core inflammatory “wiring” was overall similar across patients (and if the ultimate life and death outcome were due to a differential activation of this “wiring” due to different initial conditions or patient-specific characteristics. The first 4 principal components driving inflammation in survivors and non-survivors were IL-6, IL-10, MIP-1β, and IL-8.

We next sought to gain further insights into how the dynamic inflammatory responses of TBI survivors and non-survivors might differ using Principal Component Analysis (PCA), with the hypothesis that PCA would identify a core inflammation program, and that principal characteristics/drivers inferred by PCA would differ between survivors and non-survivors. We have previously utilized this approach to suggest primary drivers of inflammation in mice subjected to experimental trauma/hemorrhagic shock (Mi et al., 2011), in rats undergoing sepsis in the presence or absence of hemoadsorption as an experimental treatment (Namas et al., 2012), in swine undergoing experimental endotoxemia (Nieman et al., 2012), and in human pediatric acute liver failure (Azhar et al., 2013) and trauma (Namas et al., 2016a). We first carried out PCA of the full CSF time course data in all TBI patients, which identified the first principal component as a linear combination of TNF-α, MIP-1α, MIP-1β, IL-8, IL-10, and IL-6 (Figure 3A).

This analysis suggested a core, dynamic inflammation program characterized by chemokines (MIP-1α, MIP-1β, IL-8), pro-inflammatory cytokines (TNF-α, IL-6), and anti-inflammatory cytokines (IL-10). This analysis also supports the well-established roles for IL-6, IL-8, and IL-10 in TBI (Bell et al., 1997; Morganti-Kossman et al., 1997; Maier et al., 2001; Woodcock and Morganti-Kossmann, 2013).

We next carried out this analysis separately on survivors and non-survivors, in an attempt to discern whether survival and death were driven by a differential activation of this core inflammatory program, due to different initial conditions or patient-specific characteristics. The separate PCA conducted on time course CSF data from survivors and non-survivors suggested similar primary characteristics of inflammation in these patient sub-groups: IL-6, IL-10, MIP-1β, and IL-8, the first 4 components, were shared by both survivors and non-survivors (Figures 3B,C).

### DyBN inference suggests differential activation of the inflammatory network in TBI survivors and non-survivors

We next hypothesized that, despite sharing similar primary characteristics, TBI survivors, and non-survivors would differ on the basis of dynamic networks of inflammation. Biological networks often exhibit properties of “switches,” in which inferred positive and negative feedback structures are hypothesized to lead to different biological programs depending on the state of the network (Eungdamrong and Iyengar, 2004; Ferrell, 2013). In multiple prior studies of the inflammatory response in humans, we have suggested that “central nodes” in DyBN networks are those nodes exhibiting self-feedback and also cross feedback with other self-feedback nodes (Azhar et al., 2013; Zaaqoq et al., 2014; Almahmoud et al., 2015a,b; Abboud et al., 2016; reviewed in Namas et al., 2015).

**Figure 4:**
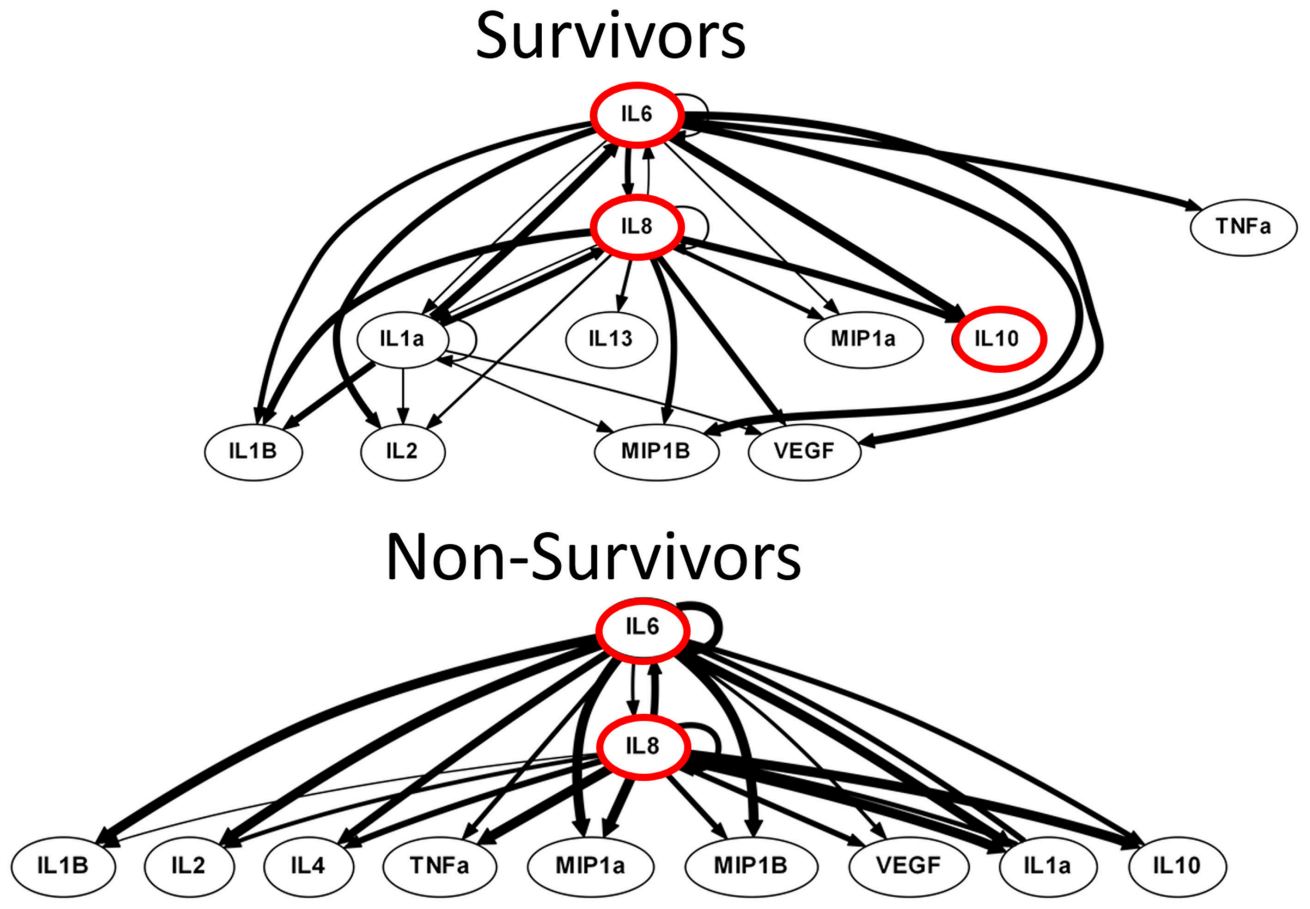
**Non-survivors DyBN was characterized by a bimodal switch state, conferring a homogeneous inflammatory response**. Both survivor and non-survivor inflammatory networks shared the IL-6 and IL- 8 as central nodes, an observation which is supported by elevated levels of both IL-6 and IL-8 in the CSF analysis. Importantly, a key difference was that the non-survivors system was characterized by a bimodal inflammatory switch state that confers an inevitable and homogeneous inflammatory response while the survivors had a tri-modal structure. The non-survivors network displayed all outputs shared between IL-6 and IL-8, irrespective of switch state. Such a network characteristic is congruent with a unimodal inflammatory phenotype, in this case a pathological type which ultimately leads to mortality. Notably, the survivors system contained the addition of a unique central node, IL-1α, which seemingly conferred a selective advantage for these patients. Thus, a DyBN inference showing an inflammatory network capable of mounting many diverse responses is a defining feature of survival.

DyBN inference based on time course data from survivors and non-survivors suggested the presence of a dynamic inflammatory response encompassing several central nodes connected to several outputs. Similar to PCA, DyBN inference of data from both survivors and non-survivors suggested that the cytokine IL-6 and the chemokine IL-8 were central nodes in the CSF post-TBI, exhibiting self-feedback and affecting each other as well as downstream nodes. In addition, DyBN inference in data from TBI survivors suggested a third mediator, IL-1α, involved in a hypothetical switch state (Figure 4).

### A mechanistic model based on ordinary differential equations suggests differences in microglial and damage responses in TBI survivors vs. non-survivors

The foregoing data-driven modeling suggested two key hypotheses centered on the cytokine IL-6. First, both PCA and DyBN identified IL-6 as a central characteristic and dynamic network node, respectively, which is substantiated by multiple prior studies (Bell et al., 1997; Morganti-Kossman et al., 1997; Maier et al., 2001; Woodcock and Morganti-Kossmann, 2013). A more specific hypothesis, derived from DyBN inference, is the presence of positive feedback behavior in IL-6. To test these hypotheses, and sepcifically the importance of IL-6 positive feedback in driving survival or death, we sought to use mechanistic mathematical modeling.

We constructed a mechanistic model based on the PCA and DyBN studies, leveraging prior experience with modeling acute inflammation mechanistically in the contexts of sepsis, endotoxemia, traumatic injury, and wound healing (Kumar et al., 2004, 2008; Chow et al., 2005; Day et al., 2006; Reynolds et al., 2006; Mi et al., 2007; Daun et al., 2008; Li et al., 2008; Torres et al., 2009; Nieman et al., 2012; Brown et al., 2015). A key feature of these prior mechanistic models is the forward feedback loop of inflammation 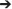 tissue damage/dysfunction 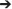 inflammation (An, 2014; Namas et al., 2015), which we hypothesize plays out in the present context in the form of IL-6 positive feedback.

**Figure 5:**
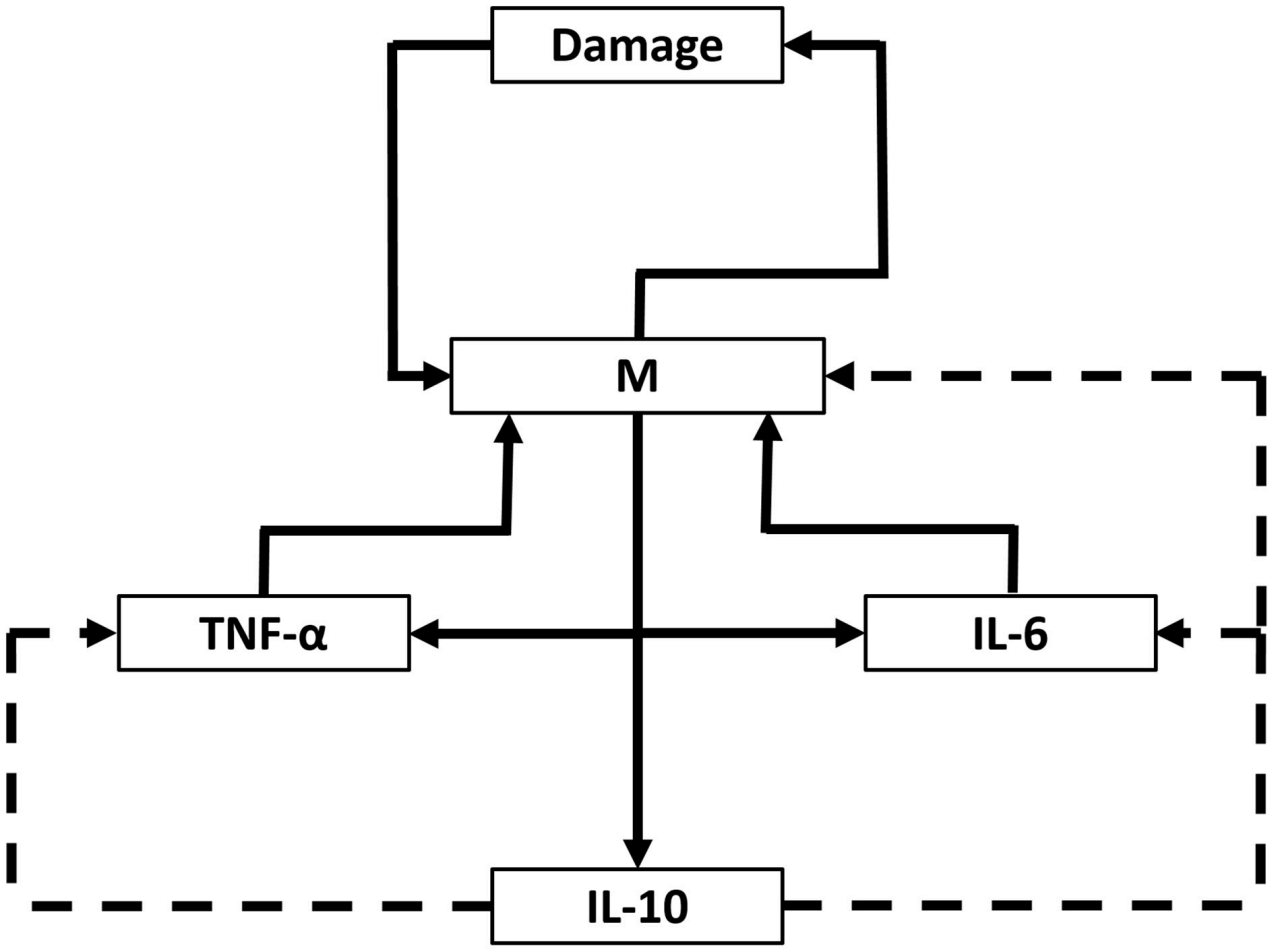
**Schematic of ODE model**. A mechanistic model using ordinary differential equations (ODE), based on core interactions inferred from PCA and DyBN and the hypothesis that inflammation drives tissue damage/dysfunction, release of DAMPs, and further inflammation in a positive feedback cycle centered on the cytokine IL-6. D, damage/dysfunction/DAMPs; M, activated inflammatory cells/microglia; C, chemokine.

To test this hypothesis, we constructed the ODE model shown schematically in Figure 5, in which IL-6 feeds back upon its own production/release by activated brain microglia (as well as potentially by astrocytes and neurons) as a function of damage/DAMPs (such as S100B), whose release is induced by injury (Woodcock and Morganti-Kossmann, 2013). We included chemokines in the model as well, based on PCA and DyBN inference, and due to their role in activating local inflammatory cells as well as attracting exogenous inflammatory cells into the brain as the blood-brain barrier breaks down following TBI (Pearn et al., 2016). Finally, the model included TNF-α and IL-10 as prototypical pro- and anti-inflammatory mediators, respectively.

**Figure 6:**
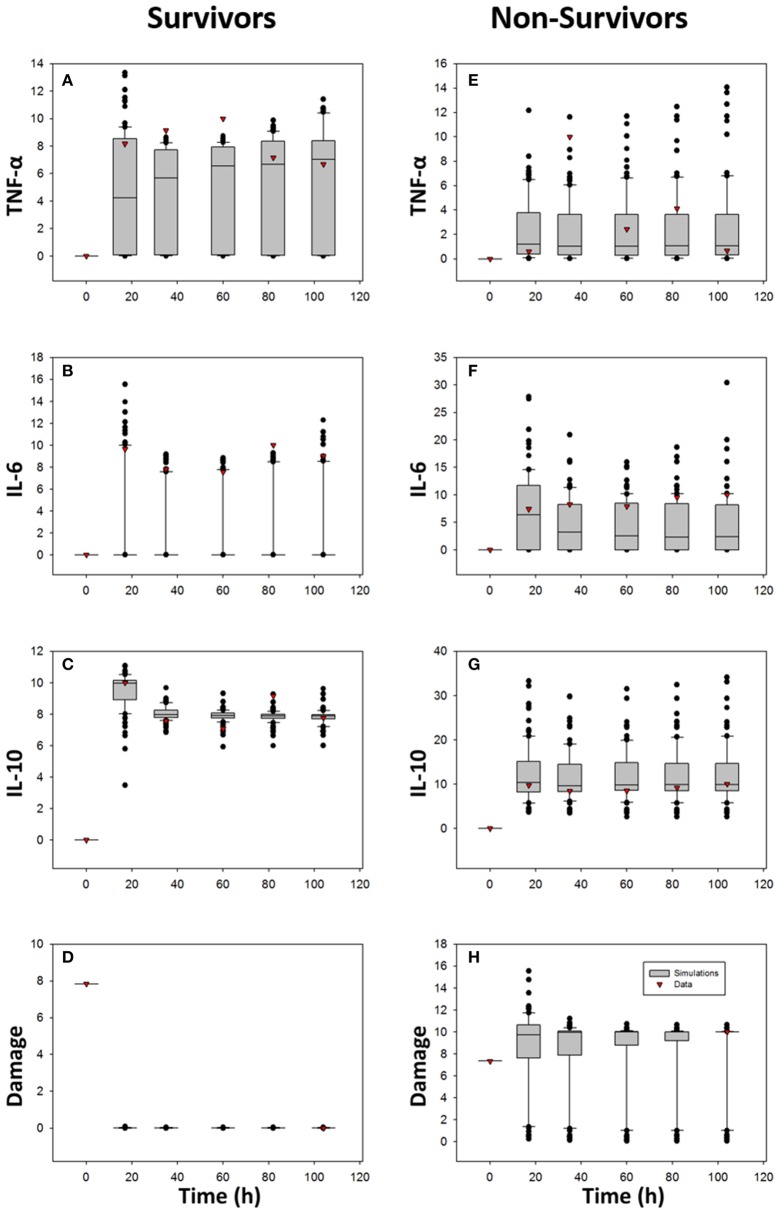
**Comparison of ODE model predictions vs. data**. The ODE model described schematically in Figure 5 and detailed in the Section Materials and Methods was fit separately to the CSF data of TBI survivors and non-survivors, respectively. One-hundred simulations were performed, of which 25 were outliers relative to the data and so were omitted (resulting in a final total of 75 simulations). The data are shown as box and whisker plots, with black symbols representing simulated data at the given time point, and the red symbols representing actual data at those time points. Damage variable data are scaled to the GCS and ultimate survival or death outcome as described in the Section Materials and Methods. **(A,E)** TNF-α; **(B,F)** IL-6; **(C,G)** IL-10; **(D,H)** Damage.

We used ordinary differential equations (ODE) to generate this fairly abstract, lumped parameter model (see Table 2 for model parameters); the model building and parameterization process is detailed in the Section Materials and Methods. We hypothesized that fitting the TBI ODE model to data for survivors separately from non-survivors—and creating ensembles of survivor-specific and non-survivor-specific models followed by an interrogation of the changes in parameter values and initial conditions required to account for these fitted model (Prince et al., 2006; Namas et al., 2013)—would allow us to gain insights into how a similar dynamic network of interactions could lead to such divergent outcomes following TBI. Accordingly, we fit the ODE model to data as described in the Section Materials and Methods, leading to an ensemble of simulations depicted in Figure 6 relative to the CSF levels of TNF-α, IL-6, and IL-10 in survivors and non-survivors, respectively. In this study, we ran 100 simulations of which 25 were removed as outliers relative to the data (leaving 75 simulations total). Overall, both survivor-specific and non-survivor-specific models fit relatively well to the IL-10 and TNF-α data, with the fits to IL-6 data being poorer (Figure 6). The Damage variable fits are plausible given a GCS score-scaled starting value and a final value scaled to either survival (damage = 0, the minimal value) or death (damage = 10, the maximal value).

We next rank-ordered the fold change between survivors' mean estimated parameter values and ones from non-survivors' based on 75 simulations, i.e., ratio = $\frac{Survivor}{Non-survivor}$ (see Table 3). This analysis of ODE model simulations suggested that the key differences between TBI survivors and non-survivors were as follows:

- A nearly 6-fold increase in the value of *d0*, the rate of damage/DAMP production by activated inflammatory cells/microglia in response to a given magnitude of injury, in non-survivors over survivors;
- A nearly 4-fold increase in the value of *init_M*, the initial value for inflammatory cells/microglia, in non-survivors over survivors;
- An approximately 3.5-fold increase in the value of *d1*, the decay rate of damage/DAMPs, in survivors over non-survivors; and
- An approximately 2.7-fold increase in the value of *i0*, the rate of IL-10 production by activated inflammatory cells, in survivors over non-survivors.

**Table 3:** **Fold changes (survivors:non-survivors) in parameter values from 75 ODE simulations**.

**Parameter**	**Survivor/non-survivor**
d1	3.538564
i0	2.701914
m6	1.235946
m8	1.209746
m7	1.209198
t0	1.163035
m1	1.13622
m5	1.120647
b0	1.111772
c2	1.091208
m9	1.084663
c0	1.071601
init_C	1.045688
b2	0.996189
m3	0.976978
c1	0.958686
b1	0.947126
i1	0.861608
t2	0.86098
m4	0.841159
m2	0.822942
t1	0.759086
m0	0.715864
init_M	0.263824
d0	0.17567

Thus, our simulations suggest that key determinants of whether or not a given TBI patient survives or succumbs to his/her injuries relate to the baseline levels of activated microglia at the time of injury, the degree to which microglia produce DAMPs in response to injury, the degree to which damage is healed or DAMPs are cleared, and the degree to which microglia produce IL-10 or other anti-inflammatory mediators.

## Discussion

In the present study, we sought to determine if the inflammatory response induced by injury, assessed in the form of trajectories of CSF inflammatory mediators analyzed using *in silico* methods, would shed insight into why some TBI patients survive and others die. By collecting serial CSF samples, and generating data-driven and mechanistic models of inflammation in a small group of TBI patients, we were able to segregate those who would survive the traumatic injury from those who would go on to die.

A central observation of this study was that the GCS alone could not predict mortality in TBI patients. Patients presenting with a similar degree of injury were destined for disparate clinical outcomes. This finding is, of course, not unique to this study, but has been cited by many and points to the inutility of the GCS as a tool for predicting patient outcomes (Gao and Zheng, 2015). Moreover, this finding highlights the difficulty of using clinical observations and physical examination in the post-TBI setting to stratify patients for outcomes. This further suggests underlying causes such as genetic predispositions and aberrant biological processes—such as differences in the inflammatory response to brain injury—must be considered in assessing the clinical course of TBI patients.

### Insights from statistical analysis and data-driven modeling

We hypothesized that underlying inflammatory processes and their downstream immunoexcitotoxic effects would be imperative to driving pathology following TBI. In support of this hypothesis, survivors could be differentiated from non-survivors based on levels of six inflammatory mediators in the CSF, similar to multiple prior studies (Bell et al., 1997; Morganti-Kossman et al., 1997; Maier et al., 2001; Woodcock and Morganti-Kossmann, 2013), albeit with levels that were dynamically changing in nature and present mostly at low levels. Thus, the CSF inflammatory mediator analysis highlights the dynamic complexity of the post-TBI inflammatory response, and suggests the need for dynamic, non-linear modeling approaches to capture key features of inflammation in these patients.

One such data-driven approach, DyBN, has been used recently in the setting of post-traumatic inflammation to successfully differentiate a group of blunt trauma survivors and non-survivors (Abboud et al., 2016). In the present study, DyBN inferred both conserved and divergent dynamic networks in TBI survivors and non-survivors, respectively. The inferred dynamic CSF inflammation networks of both survivors and non-survivors shared the cytokine IL-6 and the chemokine IL-8 as central nodes, an observation which is supported by significantly elevated levels of both IL-6 and IL-8 in the CSF in both this and prior studies (Bell et al., 1997; Morganti-Kossman et al., 1997; Maier et al., 2001; Woodcock and Morganti-Kossmann, 2013). Importantly, a key difference was that the non-survivors system was characterized by a bimodal inflammatory switch state that confers an inevitable and homogeneous inflammatory response. A key network characteristic was the suggestion that output nodes are shared between IL-6 and IL-8, irrespective of which central node (IL-6 or IL-8) dominates the “switch.” Such a network characteristic is congruent with a unimodal inflammatory phenotype, in this case a pathological type which ultimately leads to mortality.

Conversely, the dynamic network inferred in survivors was characterized by IL-6, IL-8, and an additional central node, IL-1α, which seemingly conferred a selective advantage for these patients. In the inflammatory network of survivors, a three-way switch state with variable output characteristics suggests that patients who would go on to survive were capable of recruiting additional inflammatory pathways, through which a survival advantage was conferred. Thus, we hypothesize that a dynamic inflammatory network capable of mounting variable responses is a hallmark of survival. Interestingly, IL-1α is both a cytokine and a DAMP that is induced in the context of injury (Namas et al., 2015) that was recently inferred through modeling to be a central part of the injury response to both peripheral tissues (Starzl et al., 2015) and nerves (Vasudeva et al., 2015). Furthermore, these findings may also suggest a host-protective role for IL-1α and possibly the NLRP3 inflammasome pathway (Davis et al., 2011) following TBI, which should be studied further.

### Insights from mechanistic modeling

To expand upon the insights and hypotheses generated from data-driven modeling, we generated an ODE model that was based on a core set of interactions discerned from PCA and DyBN, which in turn were derived from time courses of CSF inflammatory mediators as a proxy for TBI-induced inflammation occurring in the injured brain. Thus, the present study constitutes a progression from data to data-driven modeling to mechanistic modeling, which we have described as a rational means by which to progress from data to knowledge (An et al., 2012). We demonstrated the utility of this general approach previously, in the context of experimental endotoxemia in swine, in a study in which we used time course data and PCA to yield a two-compartment ODE model of acute inflammation (Nieman et al., 2012). In the present study, this approach was expanded to include DyBN, in order to go beyond principal characteristics to a delineation of putative feedback loops and central network nodes.

The reduced ODE model we generated was on the order of prior ODE models of acute inflammation (Kumar et al., 2004; Day et al., 2006; Reynolds et al., 2006). While such reduced models are often of predominantly theoretical utility rather than being quantitatively predictive, we nonetheless sought to parameterize the TBI model with time course of CSF data from TBI survivors vs. non-survivors. Our goal was to generate an ensemble of models differing only in the values of model parameters and initial conditions, but with identical model structure and similar fit to data, and then to leverage an approach we have described previously (Prince et al., 2006; Namas et al., 2013), in which we compared the values of these parameters in TBI survivors and non-survivors.

Given the relatively abstracted nature of our ODE model, certain formalisms that are likely not biologically realistic were employed. A key focus of the model was the inferred positive feedback behavior of IL-6. In order to obtain reasonable model fits to the data, we used the sixth power of M in the IL-6 equation to account for this positive feedback behavior because our experiments with lower powers gave worse fitting results (data not shown). Though this is likely not biologically plausible, it is a reasonable formalism given the simplicity of our model and the fact that the positive feedback behavior of IL-6 likely involves multiple indirect mechanisms that are not modeled.

Despite such limitations inherent in the process of abstraction, the ensemble modeling approach we utilized led to several plausible hypotheses regarding the differences underlying TBI survivors and non-survivors. These hypotheses center on an elevated number of activated inflammatory cells/microglia at baseline in non-survivors vs. survivors; an elevated production of DAMPs by non-survivor inflammatory cells in response a similar magnitude of injury; and, conversely, a higher clearance rate for DAMPs and a higher anti-inflammatory response (assessed as CSF IL-10) in survivors vs. non-survivors. Future studies are required in order to both validate these hypotheses and refine the ODE model to account more explicitly for the biological interactions abstracted in the current model. Clearly, these hypotheses are related to the structure of the model we utilized, which in turn is based on a core hypothesis that our group has developed over several years regarding the positive feedback loop of inflammation 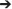 damage/dysfunction 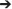 inflammation (An, 2014; Namas et al., 2015). There may well be alternative hypotheses to explain the evolution of inflammation in TBI, but this positive feedback-based hypothesis does concur with the inferred positive feedback behavior of a key inflammatory mediator, the cytokine IL-6.

This study was primarily limited by the small number of patients enrolled and the number of data points collected. This study could also be improved by utilizing a stringent case-controlled 1:1 matching protocol to control for confounding clinical factors between patients. We additionally recognize the difficulty of obtaining serial CSF samples in a clinical setting, and suggest further analyses of blood serum and CSF should be utilized to evaluate the utility of inflammatory mediators circulating systemically. Lastly, this study was of course limited in the fact that we could not control for the variability in each patient's course of treatment.

## Conclusions

The present study highlights a potential pathway by which to go from dynamic data via data-driven modeling to mechanistic modeling in a complex human inflammatory disease. While further studies are needed in order to validate key predictions, the present study may point to both novel mechanistic insights and clinically translational applications.

## Author contributions

AA and QM, Substantial contributions to the conception of the work, computational analysis, interpretation of data, and manuscript text; AP and DO, Substantial contributions to the design of the clinical data collection and analysis; MB and GC, Substantial contributions to the computational analysis and interpretation of data; YV, Substantial contributions to the conception of the work, computational analysis, interpretation of data, and critical revisions of the manuscript text for important intellectual content.

### Conflict of interest statement

The authors declare that the research was conducted in the absence of any commercial or financial relationships that could be construed as a potential conflict of interest.

## Abbreviations

CBF: cerebral blood flow
CSF: cerebrospinal fluid
DAMP: damage-associated molecular pattern molecule
GCS: Glasgow Coma Scale
GOS: Glasgow Outcome Scale
IL: interleukin
MIP: macrophage inflammatory protein
TBI: traumatic brain injury
TNF-α: tumor necrosis factor-α
VEGF: vascular endothelial growth factor.
